# Congestive Hepatopathy: A Review of the Literature

**DOI:** 10.7759/cureus.58766

**Published:** 2024-04-22

**Authors:** Rakahn Haddadin, Christopher Aboujaoude, George Trad

**Affiliations:** 1 Internal Medicine, MountainView Hospital, Las Vegas, USA

**Keywords:** systolic heart failure, liver function, right-sided heart failure, elevated liver associated enzymes, congestive hepatopathy

## Abstract

Congestive hepatopathy (CH), stemming from compromised hepatic venous flow or heightened intrahepatic pressure, represents a significant consequence of cardiovascular conditions like congestive heart failure (CHF). This review of literature encapsulates the core aspects of this condition, characterized by hepatic congestion, cellular injury, and impaired liver function. Diagnostic challenges arise due to symptoms mirroring primary liver diseases. Management revolves around addressing the underlying cause and mitigating fluid retention. This review of literature provides a snapshot of CH's complexity, emphasizing its clinical implications and the need for comprehensive understanding in clinical practice.

## Introduction and background

Congestive hepatopathy (CH), a complex hepatic condition resulting from compromised hepatic venous flow or increased pressure within the liver, is predominantly associated with congestive heart failure (CHF) but can stem from various cardiovascular and hepatic disorders [1]. Pressure is transmitted from elevated cardiac pressures to central veins of the liver, which over time causes presinusoidal dilation, decreased hepatic artery blood flow, and decreased arterial oxygen saturation [2]. The hallmark of this condition lies in hepatic congestion due to impaired venous outflow, leading to liver injury and functional impairment [3]. The results of all the congestion and backing up of pressure is possible fibrosis, cirrhosis, and even hepatocellular carcinoma (HCC) [4]. Characterized by a spectrum of nonspecific symptoms, including abdominal discomfort, hepatomegaly, jaundice, and abnormal liver function tests, CH poses diagnostic challenges often overlapping with primary liver diseases [4]. The pathophysiology involves chronic elevation in hepatic venous pressure, inducing hepatocellular damage, sinusoidal dilation, and subsequent fibrosis [5].

Diagnostic modalities, including imaging studies and laboratory tests, aid in confirming hepatic congestion and associated alterations [6]. Management primarily revolves around addressing the underlying cause, optimizing treatment for the primary condition driving hepatic congestion, and mitigating fluid retention using diuretics [5]. This comprehensive review aims to provide a detailed synthesis of the current understanding of CH, encompassing its pathophysiology, clinical presentation, diagnostic approaches, management strategies, and implications in clinical practice.

## Review

Epidemiology of CH in chronic heart failure (CHF)

CH, often referred to as cardiac hepatopathy, is intricately linked to chronic heart failure (CHF) due to the complex interplay between impaired cardiac function and hepatic hemodynamics. The association between CHF and CH is profound, with CHF serving as a primary driver of elevated liver blood vessel pressure [7].

Association with right-sided heart failure (HF) and underlying causes

Right-sided HF is a significant precursor to CH, and several underlying conditions can precipitate this cardiac pathology. Notable considerations include severe tricuspid regurgitation, severe pulmonary hypertension, constrictive pericarditis, congenital heart disease, and late-stage cardiomyopathies [7]. These conditions exert increased pressure on the right side of the heart, leading to impaired cardiac function and subsequent hepatic congestion.

Prevalence and correlation with CHF

Approximately 20-30% of CHF cases progress to CH, highlighting the clear correlation between these two conditions [8]. This statistic underscores the substantial clinical burden of CH within the CHF population and emphasizes the importance of early detection and intervention.

Risk factors for CHF

Several independent risk factors contribute to the development of CHF, further amplifying the risk of CH. These risk factors encompass demographic, lifestyle, and medical variables, including male sex, poor socioeconomic status, physical inactivity, tobacco use, higher body mass index (BMI), and a history of diabetes mellitus, hypertension, valvular heart disease, and/or coronary heart disease [9]. Each of these factors independently increases the likelihood of developing CHF, thereby elevating the risk of subsequent hepatopathic complications.

Mitigation strategies and early intervention

Early identification of CHF and its associated risk factors is paramount for mitigating the risk of developing CH. Once CHF is diagnosed, targeted interventions aimed at lifestyle modifications, cardiac care, and management of comorbidities can help reduce the likelihood of hepatic complications [10]. By addressing underlying risk factors and implementing timely interventions, healthcare providers can effectively manage CHF and mitigate the progression to CH.

Impact on healthcare policies

An improved understanding of the epidemiological trends surrounding CH informs targeted interventions and shapes healthcare policies aimed at addressing its multifactorial nature. By recognizing the interplay between CHF and CH, policymakers can allocate resources effectively, implement preventive measures, and develop strategies to improve patient outcomes [10].

In summary, the epidemiology of CH underscores its close association with CHF and highlights the importance of early intervention and risk factor management. By addressing underlying cardiac pathology and implementing targeted interventions, healthcare providers can mitigate the risk of CH and improve patient outcomes within the CHF population.

Pathophysiology of CH in CHF

CH, a consequence of CHF, arises from the intricate interplay between hemodynamic alterations and liver function [4]. CHF sets off a cascade of pathophysiological mechanisms that profoundly impact the structure and function of the liver [11].

Increased central venous pressure and sinusoidal congestion

The initial event in the pathogenesis of CH is the elevation of central venous pressure due to decreased cardiac output and systemic congestion, characteristic of CHF [11]. This heightened pressure is transmitted retrogradely to the liver through the hepatic veins, impeding normal blood flow. As a result, sinusoidal congestion occurs, defined as elevated central venous pressure causing congestion within the liver sinusoids, thereby impairing blood drainage from the liver [11]. This stasis triggers dilation of the hepatic sinusoids and congestion of the hepatic microvasculature, further impeding oxygen and nutrient delivery to hepatocytes [12].

Impaired hepatocyte function and inflammatory pathways

Prolonged congestion and reduced perfusion compromise hepatocyte function, impairing the liver's ability to synthesize proteins, metabolize toxins, and perform other essential functions [13]. This hepatocellular dysfunction leads to the activation of inflammatory pathways. Stagnant blood flow and hypoxia within the liver trigger the release of pro-inflammatory cytokines and oxidative stress. This inflammatory milieu exacerbates hepatocellular damage and promotes fibrogenesis [12].

Fibrogenesis and structural changes

Persistent injury prompts hepatic stellate cells to transform into myofibroblasts, initiating fibrogenesis and collagen deposition [12]. Fibrosis ensues, leading to architectural distortion and compromise of the liver's structural integrity. Fibrotic changes increase intrahepatic resistance, contributing to elevated portal pressure. Portal hypertension arises, leading to collateral vessel formation and complications, such as ascites and varices.

Inflammatory and fibrogenic mediators

The inflammatory and fibrogenic mediators involved in the pathogenesis of CH include cytokines, such as tumor necrosis factor-alpha (TNF-alpha), interleukin-6 (IL-6), and transforming growth factor-beta (TGF-beta). These mediators play a pivotal role in hepatocellular damage, fibrogenesis, and the progression of liver pathology [12].

Hepatic ischemia and necrosis

In severe cases of CH, prolonged hepatic congestion and compromised blood flow can lead to hepatic ischemia and necrosis. Ischemic hepatocyte injury exacerbates liver dysfunction and may precipitate acute liver failure, further complicating the clinical course of CHF [12].

Interplay with systemic and cardiac dysfunction

**Figure 1:**
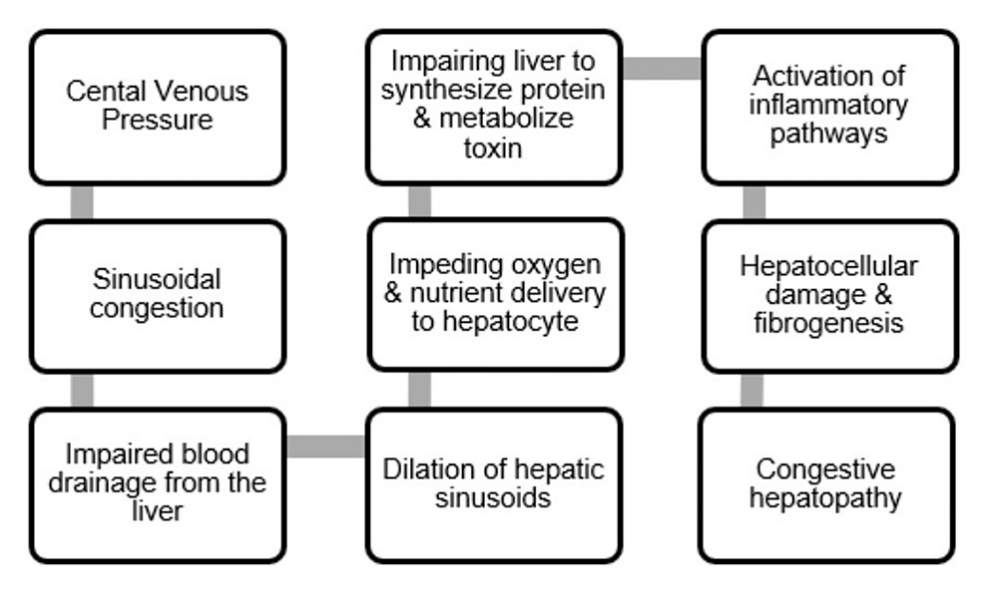
Pathophysiology of congestive hepatopathy Original figure made by the author.

The pathophysiological mechanisms underlying CH are closely intertwined with systemic and cardiac dysfunction in CHF (Figure 1). As cardiac output decreases and systemic congestion ensues, hepatic perfusion is compromised, setting the stage for the development of hepatocellular injury and fibrosis [11]. In addition, systemic inflammation and oxidative stress, characteristic of CHF, further exacerbate liver pathology, contributing to the progression of CH [12].

Understanding the underlying pathophysiological mechanisms is crucial for the early detection and management of CH in patients with CHF. By targeting key pathways involved in liver injury and fibrosis, clinicians can optimize therapeutic interventions and improve outcomes for patients affected by this hepatovascular syndrome.

Fibrogenesis and structural consequences

Persistent injury within the liver microenvironment triggers a cascade of events culminating in fibrogenesis and collagen deposition [12]. Hepatic stellate cells, in response to injury, undergo activation and transform into myofibroblasts, initiating the process of fibrosis [12]. This pathological remodeling of the liver architecture leads to architectural distortion and compromise of its structural integrity. As fibrosis progresses, intrahepatic resistance increases, contributing to elevated portal pressure [14].

Portal hypertension and complications

Elevated portal pressure, a consequence of fibrotic changes, precipitates portal hypertension, heralding the formation of collateral vessels and ensuing complications, such as ascites and varices [14]. Collateral vessel formation serves as a compensatory mechanism to divert blood flow away from areas of increased resistance, thereby mitigating the effects of portal hypertension. However, this adaptive response can lead to the development of varices, predisposing individuals to life-threatening complications, such as variceal bleeding. Ascites, characterized by the accumulation of fluid in the peritoneal cavity due to increased portal vein pressure, further exacerbate the clinical burden of CH [5].

Functional decline of the liver

With progressive fibrosis and ongoing hepatocellular injury, the liver's synthetic, metabolic, and detoxifying functions become compromised [15]. Hypoalbuminemia ensues due to impaired synthesis of albumin, contributing to decreased oncotic pressure and fluid retention. Coagulopathy may develop as hepatic synthetic dysfunction leads to impaired production of clotting factors. Jaundice, resulting from the accumulation of bilirubin due to impaired liver function, manifests as yellowing of the skin and eyes. In addition, susceptibility to infections increases as impaired immune function compromises the liver's ability to clear pathogens and toxins [15].

Clinical manifestations and symptomatology

Patients with CH may present with a spectrum of symptoms reflecting the diverse pathological consequences of hepatic congestion and dysfunction [16]. Hepatomegaly, attributable to liver enlargement secondary to congestion, may be palpable on physical examination [5]. Jaundice, a hallmark of hepatic dysfunction, manifests as yellowing of the skin and sclera due to bilirubin accumulation [5]. Ascites, resulting from increased portal vein pressure, present as abdominal distension and discomfort. Abdominal pain may arise from the distension of the liver capsule and stretching of the hepatic parenchyma. Fatigue and weakness ensue from decreased liver function and overall poor circulation, impacting the individual's quality of life. Nausea and vomiting may occur as a consequence of liver congestion affecting bile flow and digestion [2]. Importantly, these symptoms may occur in the absence of ascites, highlighting the diverse clinical presentation of CH [2].

Understanding the underlying pathophysiological mechanisms and clinical manifestations of CH is crucial for timely diagnosis and targeted interventions aimed at mitigating disease progression and improving patient outcomes. By addressing the diverse clinical manifestations and complications associated with CH, healthcare providers can optimize patient care and enhance the quality of life for affected individuals.

Diagnosis of CH in CHF

Diagnosing CH in the context of CHF requires a comprehensive approach encompassing medical history, physical examination, laboratory tests, and imaging studies. Timely and accurate diagnosis is essential for initiating appropriate management strategies and optimizing patient outcomes [2].

Medical history and physical examination

Clinicians should begin by obtaining a detailed medical history, with particular emphasis on symptoms suggestive of hepatic dysfunction and underlying heart conditions [2]. Common symptoms include hepatomegaly, jaundice, abdominal discomfort, and fluid accumulation in the abdomen (ascites) [2]. A thorough physical examination focusing on signs of liver enlargement (hepatomegaly), jaundice, and ascites is crucial for identifying clinical clues suggestive of CH [2].

Laboratory tests

Blood tests play a pivotal role in the diagnostic workup of CH, revealing abnormalities in liver function and coagulation parameters. Elevated liver enzymes, such as aspartate transaminase (AST) and alanine transaminase (ALT), may indicate hepatocellular injury secondary to hepatic congestion [2]. In addition, elevated lactate dehydrogenase (LDH) levels, typically 10-20 times the upper limit of normal, may be observed following hemodynamic deterioration in CHF [2]. Bilirubin levels may also be elevated, although to a lesser extent compared to other liver pathologies, with mean levels typically lower than 6 mg/dL [17]. Elevated bilirubin levels beyond the mean may suggest possible progression to acute liver failure [18].

Brain natriuretic peptide (BNP) levels can serve as a useful adjunctive tool in differentiating between cardiac ascites and cirrhotic ascites in uncertain cases [5]. Elevated serum and ascites BNP levels (>364 pg/mL) have demonstrated high sensitivity (98%) and specificity (99%) in predicting cardiac causes for ascites, aiding in accurate diagnosis and management [19].

Imaging studies

Imaging studies play a crucial role in confirming the diagnosis of CH and identifying potential complications. Abdominal ultrasound serves as the first-line imaging modality due to its accessibility and ability to assess liver morphology and vascularization [2]. Ultrasound findings may include liver enlargement, increased echogenicity (indicating fatty changes), and signs of portal hypertension such as splenomegaly and ascites [20].

Computed tomography (CT) and magnetic resonance imaging (MRI) are preferred modalities for better morphological characterization of the liver and identification of abnormal kinetics associated with CH [2]. These imaging techniques provide detailed information about liver architecture, hepatic blood flow, and the presence of complications, such as ascites and varices. In addition, echocardiography can help assess cardiac function and evaluate the potential contribution of cardiac causes to elevated liver function enzymes [21].

By leveraging a multidimensional diagnostic framework, healthcare providers can accurately identify and characterize CH, facilitating timely intervention and optimized management strategies for patients with CHF-associated liver pathology.

Treatment strategies for CH in CHF

Managing CH in the context of CHF requires a multidisciplinary approach aimed at addressing both the underlying cardiac pathology and hepatic congestion. While there is no direct therapy specifically targeting CH, treatment primarily revolves around managing the underlying condition causing liver congestion [2].

Inclusion of liver function tests in HF guidelines

Given the significant association between liver dysfunction and HF prognosis, guidelines from the American College of Cardiology and the European Society of Cardiology recommend the inclusion of liver function tests in all patients with HF during diagnostic workups [22,23]. Liver enzymes, such as bilirubin, alkaline phosphatase, gamma-glutamyl transferase, and albumin, along with scores like the Model for End-Stage Liver Disease (MELD), can provide valuable prognostic information in HF patients [24]. Elevated liver enzymes and MELD scores correlate with worse outcomes, highlighting the importance of monitoring liver function in CHF patients [25].

HF management

Optimizing HF management remains the cornerstone of treatment for CH. Medications such as diuretics play a crucial role in reducing fluid retention and alleviating symptoms of congestion, thereby easing the pressure on the liver [10]. ACE inhibitors, beta-blockers, and other medications may also be prescribed to improve heart function, reduce workload, and manage blood pressure [10]. However, clinicians must exercise caution with medications that may exacerbate liver dysfunction, particularly in the context of cirrhosis [2].

Adjunctive therapies

Limited data exist on the use of adjunctive therapies for CH. N-acetylcysteine, for example, has been proposed as a potential treatment option due to its ability to mitigate vascular filling and minimize passive hepatic congestion [2]. In addition, dobutamine, with its inotropic and vasodilating effects, may help alleviate hepatic congestion caused by cardiac etiologies [26]. However, further research is needed to elucidate the efficacy and safety of these adjunctive therapies in the management of CH.

Management of complications

In cases where complications such as ascites or jaundice arise, specific interventions may be required. Diuretics, paracentesis (draining of ascitic fluid), or albumin infusions may be utilized to manage ascites and alleviate symptoms [2]. Treatment for jaundice involves addressing the underlying cause and providing supportive care to improve liver function. In severe and refractory cases, advanced interventions such as implantable devices (e.g., cardiac resynchronization therapy devices), ventricular assist devices, or heart transplantation may be considered [2]. In extreme cases with irreversible liver damage, liver transplantation could potentially be an option, albeit rare.

Multidisciplinary collaboration

Close collaboration between cardiologists, hepatologists, and other specialists is paramount in tailoring treatment strategies and optimizing outcomes for individuals with CH [2]. The multidisciplinary approach aims to alleviate symptoms, improve heart function, manage complications, and prevent disease progression, ultimately enhancing the quality of life for affected individuals.

**Figure 2:**
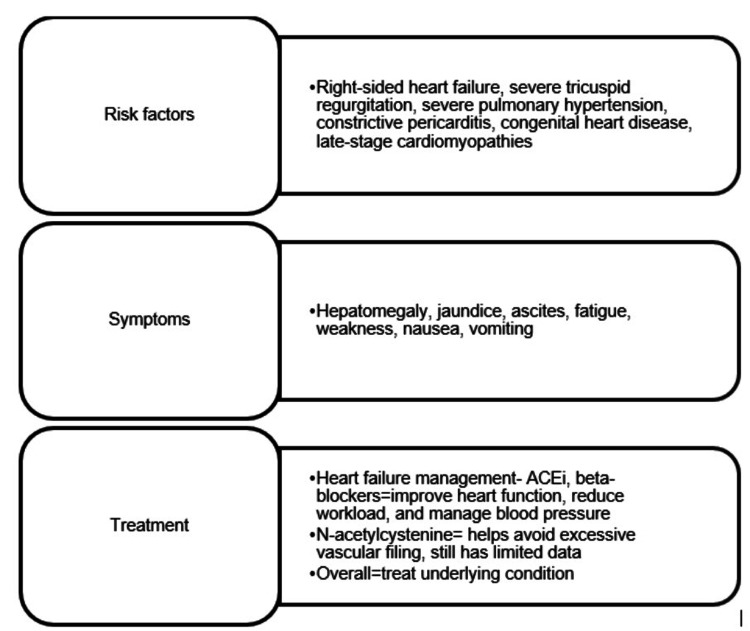
Summary of risk factors, symptoms, and treatments for congestive hepatopathy Original figure made by the author.

The management of CH in CHF necessitates a comprehensive approach encompassing HF management, adjunctive therapies, and the management of complications [27]. Close monitoring of liver function, adherence to guidelines, and multidisciplinary collaboration are essential for optimizing patient outcomes in this complex clinical scenario. The summary of risk factors, symptoms, and treatments for CH is shown in Figure 2.

## Conclusions

In reviewing the literature on CH, it is evident that this condition represents a significant consequence of compromised venous flow within the liver, commonly linked to heart failure. The comprehensive understanding of its clinical presentation, diagnosis, and treatment emphasizes the necessity for a multifaceted approach. Addressing the underlying cause, chiefly heart failure, stands as the cornerstone of effective management, involving medication, lifestyle modifications, and vigilant monitoring.

Research continually enriches therapeutic avenues, highlighting the importance of tailored interventions to alleviate hepatic congestion and associated complications like ascites or jaundice. The evolving landscape underscores the need for collaborative efforts among healthcare disciplines to refine treatment strategies, enhance patient outcomes, and pave the way for improved care paradigms in CH.

## References

[1] Weisberg IS, Jacobson IM (2011). Cardiovascular diseases and the liver. Clin Liver Dis.

[2] Ford RM, Book W, Spivey JR (2015). Liver disease related to the heart. Transplant Rev (Orlando).

[3] Hilscher M, Sanchez W (2016). Congestive hepatopathy. Clin Liver Dis (Hoboken).

[4] Myers RP, Cerini R, Sayegh R, Moreau R, Degott C, Lebrec D, Lee SS (2003). Cardiac hepatopathy: clinical, hemodynamic, and histologic characteristics and correlations. Hepatology.

[5] Fortea JI, Puente Á, Cuadrado A (2020). Congestive hepatopathy. Int J Mol Sci.

[6] Çağlı K, Başar FN, Tok D, Turak O, Başar Ö (2015). How to interpret liver function tests in heart failure patients?. Turk J Gastroenterol.

[7] Kavoliuniene A, Vaitiekiene A, Cesnaite G (2013). Congestive hepatopathy and hypoxic hepatitis in heart failure: a cardiologist's point of view. Int J Cardiol.

[8] Harjola VP, Mullens W, Banaszewski M (2017). Organ dysfunction, injury and failure in acute heart failure: from pathophysiology to diagnosis and management. A review on behalf of the Acute Heart Failure Committee of the Heart Failure Association (HFA) of the European Society of Cardiology (ESC). Eur J Heart Fail.

[9] He J, Ogden LG, Bazzano LA, Vupputuri S, Loria C, Whelton PK (2001). Risk factors for congestive heart failure in US men and women: NHANES I epidemiologic follow-up study. Arch Intern Med.

[10] Malik A, Brito D, Vaqar S, Chhabra L (2022). Congestive heart failure. StatPearls [Internet].

[11] Sessa A, Allaire M, Lebray P, Medmoun M, Tiritilli A, Iaria P, Cadranel JF (2021). From congestive hepatopathy to hepatocellular carcinoma, how can we improve patient management?. JHEP Rep.

[12] Sherlock S (1951). The liver in heart failure relation of anatomical, functional, and circulatory changes. Br Heart J.

[13] Louie CY, Pham MX, Daugherty TJ, Kambham N, Higgins JP (2015). The liver in heart failure: a biopsy and explant series of the histopathologic and laboratory findings with a particular focus on pre-cardiac transplant evaluation. Mod Pathol.

[14] Elder R, McCabe N, Hebson C, Veledar E, Romero R, Ford R (2013). Features of portal hypertension are associated with major adverse events in Fontan patients: the VAST study. Int J Cardiol.

[15] Sharma A, Nagalli S (2023). Chronic liver disease. StatPearls [Internet].

[16] Henrion J (2012). Hypoxic hepatitis. Liver Int.

[17] Fuhrmann V, Kneidinger N, Herkner H (2009). Hypoxic hepatitis: underlying conditions and risk factors for mortality in critically ill patients. Intensive Care Med.

[18] Møller S, Bernardi M (2013). Interactions of the heart and the liver. Eur Heart J.

[19] Sheer TA, Joo E, Runyon BA (2010). Usefulness of serum N-terminal-ProBNP in distinguishing ascites due to cirrhosis from ascites due to heart failure. J Clin Gastroenterol.

[20] Morales A, Hirsch M, Schneider D, González D (2020). Congestive hepatopathy: the role of the radiologist in the diagnosis. Diagn Interv Radiol.

[21] Kaneko KN, Finneman ZD, Avila PM, Lim JA, Sukpraprut-Braaten S (2022). Striking elevations in aminotransferases in a case of congestive hepatopathy without concurrent hypotension. Cureus.

[22] Allen LA, Felker GM, Pocock S (2009). Liver function abnormalities and outcome in patients with chronic heart failure: data from the Candesartan in Heart Failure: assessment of Reduction in Mortality and Morbidity (CHARM) program. Eur J Heart Fail.

[23] Ponikowski P, Voors AA, Anker SD (2016). 2016 ESC Guidelines for the diagnosis and treatment of acute and chronic heart failure: the Task Force for the diagnosis and treatment of acute and chronic heart failure of the European Society of Cardiology (ESC). Developed with the special contribution of the Heart Failure Association (HFA) of the ESC. Eur J Heart Fail.

[24] Yancy CW, Jessup M, Bozkurt B (2013). 2013 ACCF/AHA guideline for the management of heart failure: a report of the American College of Cardiology Foundation/American Heart Association Task Force on practice guidelines. Circulation.

[25] Tapper EB, Sengupta N, Bonder A (2015). The incidence and outcomes of ischemic hepatitis: a systematic review with meta-analysis. Am J Med.

[26] Kram HB, Evans T, Bundage B, Shoemaker WC (1988). Use of dobutamine for treatment of shock liver syndrome. Crit Care Med.

[27] Islam H, Puttagunta SM, Islam R (2022). Risk of stroke with mitral stenosis: the underlying mechanism, treatment, and prevention. Cureus.

